# Prevalence of Bone Density Reduction and Its Related Factors in Hemophilia Patients in South Khorasan Province in 2018

**DOI:** 10.31661/gmj.v9i0.1711

**Published:** 2020-08-27

**Authors:** Alireza Ehsanbakhsh, Ghodsiyeh Azarkar, Masood Ziaee, Ali Taghavieh

**Affiliations:** ^1^Department of Radiology, Birjand University of Medical Sciences, Birjand, Iran; ^2^Infectious Diseases Research Center, Birjand University of Medical Sciences, Birjand, Iran

**Keywords:** Bone Mineral Density, Hemophilia, Bone Mineral Density, Associated Factors

## Abstract

**Background:**

The present study aimed to determine the prevalence of bone density reduction and its associated factors in hemophilia patients in South Khorasan Province in 2018.

**Materials and Methods:**

This case-control study was conducted on all patients with hemophilia type A with medical records in Hemophilia center in South Khorasan province. A number of 57 eligible subjects who met the criteria were selected based on census method. Thereafter, 60 non-hemophilic individuals who were similar to hemophilic patients in terms of age and body mass index were selected by convenience sampling method. DXA device was used to measure bone mineral density (BMD) in two locations of femoral neck and lumbar vertebrae. Data were analyzed in SPSS software (version 19), using Chi-square, Fisher’s exact test, and independent t-test. A P-value less than 0.05 was considered statistically significant.

**Results:**

The prevalence rate of bone density reduction in spine bone in hemophilia patients was measured at 31.6% and 13.3% in non-hemophilia subjects (P=0.02); moreover, this rate in hip bone was reported as 7% and 5%, respectively (P=0.65). Relative frequency of bone density reduction in the spine and hip bone was not significantly different among hemophilia patients based on age, severity of hemophilia, vitamin D, hepatitis and smoking (P>0.05). However, a significant difference was detected in terms of body mass index (P<0.05).

**Conclusion:**

Based on the obtained results, the prevalence of bone density reduction in hemophilic patients was significantly higher, as compared to that of non-hemophilia patients. This necessitates the implementation of drastic preventive measures, prompt diagnosis, timely treatment, and appropriate therapeutic measures.

## Introduction

Hemophilia is a bleeding disorder caused by sex-related congenital deficiency occurring in about one in 1,000 births. Hemophilia A emerges due to impaired coagulation factor VIII and mutation of genes associated with factors [1] with an incidence of 1 per every 5,000-100,000 living males [2]. Recurrence of hemarthrosis which is common in patients with hemophilia triggers joint degeneration and leads to arthropathy and disability being prevalent before adulthood [3]. Frequent immobilization for the treatment of hematuria and inability caused by arthropathy are among the main risk factors for bone density reduction [4]. In the study carried out by Roshan *et al*. (2014) on hemophilia patients, the incidence rate of bone density reduction in the lumbar spine and femoral neck was reported as 2 / 45% and 31.7%, respectively [5]. The distinguishing features of osteoporosis include bone mass reduction and alteration of bone tissue architecture [6]. Moreover, it carries other complications, such as the reduction of bone mineral content along with the bone matrix in the sense that the amount of bone is reduced; however, the composition of bone remains intact [7]. Despite the absence of any obvious cause for bone density reduction in most patients, there exist many factors that make this a secondary form of the disease. These factors include a number of drug therapies and some clinical disorders, such as hyperparathyroidism, hyperthyroidism, cortisol elevation, several digestive disorders, and many other issues [8]. One of the adverse effects of bone density reduction is a decrease in the number and activity of osteoblasts. It seems that other pathologic causes, such as deficiency of gonadal hormones, bone marrow calcium, and vitamin D, exert significant effects on bone density of patients which are determined by BMD based on Dual X Ray Absorbtiometry [9]. Risk factors for bone density reduction include age, glucocorticoids use for more than three months, fracture history in first-degree relatives, low body mass index (BMI), cigarette smoking, nutritional factors, work conditions, extreme exercise, and low socioeconomic status [10-13]. Guthrie *et al*. in their study performed on women aged 46-56 years in Australia revealed that risk factors of bone density reduction include calcium intake less than 800 mg/day (52%), caffeine consumption greater than 360 mg/day (56%), exercise less than an hour and a half per week (29%), BMI below 20 (5%), current smoking rate (14%), history of smoking (23%), and existence of fracture among family members (25%). Furthermore, they reported a significant relationship between BMD and weight, and between BMI and calcium absorption [14]. Moreover, based on the results of a study, independent predictors of BMD decrease in men with hemophilia A and B were reported as physical activity and the concentration of 25-hydroxyvitamin D [15]. Due to the confusion surrounding the factors related to the reduction of bone mineral density in hemophilic patients, the present study was conducted to determine the prevalence of bone density and its associated factors in hemophilia patients in South Khorasan Province in 2018.

## Materials and Methods

###  Analysis Methodology

 This case-control study was conducted on all patients with Hemophilic type A and records in Hemophilia center in South Khorasan province. A number of 57 eligible subjects who met the criteria were selected based on census method. Thereafter, 60 non-hemophilic patients who were similar to hemophilic patients in terms of age and body mass index were selected by convenience sampling method. The inclusion criteria entailed: 1) age range of 10-50, 2) non-use of corticosteroids, 3) non-existence of liver, renal and lung disease, 4) absence of thyrotoxicosis, hyperparathyroidism, and hypercortisolism, and 5) willingness to cooperate in the study. Before the commencement of the study, approval was obtained from the Ethics Committee of Birjand University of Medical Sciences (with the code ir.Bums.REC.2017.232,3-12-2017). Thereafter, upon referral from vice-chancellor in Research Affair Department and necessary arrangements with the authorities, patient’s information was elicited from their case history in the Hemophilia Center of South Khorasan Province. All hemophilic and non-hemophilic participants were invited to bone mineral density (BMD) measurement and 5 cc of blood was extracted from them after explaining the goals and nature of the study and obtaining their written consent. Subsequently, 25-hydroxy vitamin D level was determined in clotted blood using the radioimmunoassay method in the central laboratory of Imam Reza Hospital. Based on 25 hydroxyvitamin D levels, participants were assigned to two groups of normal (greater than or equal to 20 ng/ml) and lower than normal (less than 20 Ng/ml).

While the participants were lightly clothed and were not wearing shoes, their weight was measured and recorded using a German Seca digital scale with a precision of 100 grams at 10-11 in the morning with empty urinary bladder and their height was calculated using metal meters in the standard manner. BMI was calculated using the formula: the person’s body weight (in kilograms) divided by the square of his height (in meters) and the subjects were assigned into three groups of lean (BMI≤18), normal (18> BMI>25), and overweight (BMI≥25). Dual-Energy X-ray absorptiometry (DXA) was used to measure bone mineral density (BMD). According to the suggestion of International Society for Clinical Densitometry (ISCD), BMD is measured for the purpose of diagnosis of OP in two locations of femoral neck and lumbar bone [16,17]. The method for measuring bone density (determination criteria) is expressed as T-score and Z-score; the International Society for Clinical Densitometry has suggested that young people should use Z-score instead of T-score and Z -scores less than -2 should be considered as bone mineral density reduction based on age or less than the expected range based on age and Z-score scores higher than -2 should be regarded as expected range based on age [18]. Data were analyzed in SPSS software (version 19) (Armonk, NY: IBM Corp) using the Chi-square test and Fisher’s exact test and independent t-test. A P-value less than 0.05 was considered as statistically significant.

## Results

 Out of 117 participants, 57 individuals (48.7%) were allocated to the experimental group and 60 subjects (51.3%) were assigned to the control group. The subjects in the experimental and control groups were not markedly different in terms of average age and body mass index (Table-1). Relative frequency of bone density reduction in spine bone was found to be significantly higher in the experimental group, as compared to control group (P=0.02). However, there was no significant difference in relative frequency of bone density reduction in hip bone in both experimental and control groups (P=0.65; Table-2). The mean bone density reduction in the spine bone was significantly higher in experimental group, in comparison with control group (P=0.001); however, no significant difference was detected between the two groups in case of hip bone (P=0.09; Table-3). In addition, the relative frequency of bone density reduction in spine and hip bone was not significantly different in hemophilic patients based on age, severity of hemophilia, vitamin D, and suffering from hepatitis (P>0.05). However, the highest relative frequency of bone density reduction in spine and hip bones was observed in thin patients and no bone density reduction was detected in overweight patients which was statistically significant (P<0.05; Table-4). Based on regression analysis, dependent variable alterations can be predicted by the regression model (P=0.01). In the current study, the regression model was used to predict the dependent variable alterations as depicted in Table-5. Based on the obtained results, the regression model was applicable between bone density reduction in spine bone and such factors as age, BMI, and vitamin D (P=0.01). Moreover, it was implemented for the predictability between bone density reduction in hip bone and variables, including age, BMI, and vitamin D (P=0.001). Regression analysis revealed that 13% of bone density reduction frequency in spine bone was predicted by regression model. In addition, 21% of bone density reduction frequency in hip bone was anticipated by regression model. Two factors of age and BMI predicted the frequency of bone density reduction in hip and spine bone (P<0.05); however, the level of Vitamin D was not an appropriate indicator of bone density reduction frequency of in hip and spine bone (P>0.05).

## Discussion

In the present study, the prevalence of bone density reduction among hemophilia patients was obtained as 31.6% and 7% in the spine and hip bones, respectively, and these values for non-hemophilic patients were measured at 13.3% and 5%, respectively. In the study performed by Roshan *et al*. (2014) on hemophilia patients, the prevalence of bone density reduction was reported as 45.2% in the spine bone, 31% in the left side of femoral neck, and 35.7% on the right side of femoral neck [5]. In addition, the results of the study on hemophilia patients conducted by Gerstner *et al*. (2009) indicated that 70% of patients, out of whom 43% suffered from osteopenia and 27% of patients had osteoporosis, were afflicted with BMD reduction [19]. In the study carried out by KiperUnal *et al*. (2017) on patients with moderate and severe hemophilia, the prevalence rate of bone density reduction in patients younger than 50 years old was reported as 34.8% [20]. The results of these studies were consistent with the findings of the current study. In the study conducted by Naderi *et al*. (2012), the prevalence of femoral neck osteopenia in hemophilia patients was measured at 50% [21] which was more than what obtained in the present study. Based on all the above-mentioned studies, the incidence of bone density reduction is significantly higher among hemophilia patients, as compared to non-hemophilia patients. In the study performed by Bazrafshan *et al*. (2010), the incidence of osteopenia in men referred to Densitometry center of Gorgan has been reported as 2.5 % in waist and 3.3% in femur [22]. The study conducted by Ehsanbakhsh *et al*. (2011) on the incidence of undetected spinal fractures in patients with low back pain using dual-energy X-ray absorptiometry method (DXA) for thoracic and lumbar vertebrae denoted that 13.4% of patients had normal bone mineral density in the lumbar spine, 27.9% had osteopenia, 58.7% had osteoporosis, and the prevalence of spine fracture was reported to be 39% [23]. The high rate of bone reduction in patients with hemophilia can be attributed to various factors, including reduced physical activity due to hemophilic arthropathies and fear of bleeding, pain, inflammation of the joints, vitamin D deficiency, and positivity of HCV and HIV [24,25]. In the study carried out by Naderi *et al*. (2012),45% right-shoulder joint, 30% left-shoulder, 82.5% right knee, 30% left knee, 40% right ankle, and 12.5% left ankle arthropathy was reported as prevalence rates among patients with hemophilia [21] and the above-mentioned factors inhibit individuals’ physical activity. In addition, anxiety can be another source of this problem and the results of some studies indicated that hemophilic patients develop a number of distressing factors that trigger bone mass reduction [24,25]. The results of the current study revealed that the relative frequency of bone density reduction in spine and hip bone in hemophilic patients is not significantly different based on age which is not in agreement with the results of studies conducted by Roshan *et al*. (2014) [5], Abd EL Naeem *et al*. (2016) [26], Bazrafshan *et al*. (2010), and Gerstner *et al*. (2009) [19]. This discrepancy can be attributed to differences in the number of subjects, the age range of patients, control of the disease, and physical activity. Eshghi and Morbveisi (2011) in a study conducted on children with hemophilia indicated a significant relationship between aging and BMD reduction which is inconsistent with the results of the present study. This disagreement is probably due to the age range of patients in two studies since Eshghi and Morbveisi [27] examined children with the mean age of 6.8±3.8 years and age group of 4-15 years, while the present study was carried out on the patients with the mean age of the 32.77±9.32 years within the age range of 10-50 years. Based on the results of the current study, the rate of bone density reduction in the spine and hip bones in hemophilia patients was statistically different in terms of body mass index and was reported to be significantly higher in thin patients which is in accordance with the results of studies carried out by Gerstner *et al*. (2009) [19] and Bazrafshan *et al*. (2010) [22] and incompatible with the results of studies performed by KiperUnal *et al*. (2017) [20] and Lorio *et al*. (2010) [28]. In addition, according to the results of this study, the prevalence of bone density reduction in the spine and hip bones in patients with hemophilia and hepatitis was not significantly different in term of the severity of hemophilia which is not in line with the results of SossaMelo *et al*. (2018) [29] and Lorio *et al*. (2010). The discrepancy between the results of the study conducted by Lorio *et al*. [28] and those of the present study can be attributed to the fact that their study was carried out on patients with severe hemophilia; however, hemophilic patients with mild to severe hemophilia were included in the present study. Moreover, the results of various studies have revealed no significant relationship between BMD reduction and hepatitis [5,19] which is in agreement with the results of the present study. Exposure to contaminated blood can put hemophilic patients at risk of hepatitis C (HCV) infection, chronic kidney disease, vitamin D deficiency, as well as impaired testosterone and progesterone metabolism [24]. The results of the current study indicated that the incidence of bone density reduction and osteoporosis in patients with hepatitis C is higher than that of non-hemophilic individuals; however, it is not statistically significant which can be pertinent to small sample size. In addition, the results of the current study revealed that the relative frequency of bone density reduction in spine and hip bones in hemophilic patients was not significantly different in terms of vitamin D. The results of a study performed by KiperUnal *et al*. (2017) suggested that vitamin D deficiency is common in hemophilia patients (77.5%); however, there is no statistically significant relationship between vitamin D deficiency and BMD reduction [20] which is consistent with the results of the current study. The absence of any correlation between the level of vitamin D and bone density may be attributable to secondary hyperparathyroidism which results in vitamin D reduction through bone remodeling. Equally important, based on the results of the present study, the prevalence of bone density reduction in spine and hip bone was not significantly different concerning smoking which is in accordance with the results of studies performed by KiperUnal *et al*. (2017) [20] and Roshan *et al*. (2014) [5]. On account of the recent increase in life expectancy for hemophilia patients due to preventive measures and advances in therapeutic methods, bone health and promotion of patients’ quality of life is noteworthy [30]. In addition, regarding the fact that the highest increase of bone density occurs in childhood and adolescence and reaches its maximum at the age of 25-20, which is the most important time to increase bone density and lack of activity due to pain, overprotective parents and inappropriate nutrition may gradually lead to bone density reduction [31]. Therefore, activities appropriate to their weight are recommended in order to ensure the formation of bone mass, especially in children.

## Conclusion

 Based on the obtained results, the prevalence of bone density reduction is significantly higher in hemophilia patients, as compared to normal individuals which necessitates the implementation of drastic preventive measures, prompt diagnosis, timely treatment, and appropriate therapeutic measures.

## Acknowledgment

 Our sincere appreciation and thanks go to all patients who kindly participated in the current study. Moreover, we acknowledge the valuable support of Hemophilia Society of South Khorasan. The current research was funded and supported by a research grant (Ir.Bums.REC.1396.232) from Birjand University of Medical Sciences.

## Conflict of Interest

 The authors have no conflict of interest References.

**Table 1:** Comparison of Mean Age and Body Mass Index in Subjects in Both Experimental and Control Groups

**Group** **Variable**	**Experimental **	**Control **	**P-value of Independent T-Test**
	**Mean±SD**	**Mean±SD**	
**Age**	32.9±77.32	34.8±87.40	0.20
**Body Mass Index **	21.3±49.89	22.4±68.78	0.14

**Table 2:** Comparison of Relative Frequency of Bone Density Reduction in Spine and Hip Bones in Patients in Both Experimental and Control Groups

**Decrease Bone Density** **Bone**		**Normal**	**Decrease Bone Density**	**P-value for Chi-Square Test**
		**Number (Percent)**	**Number ** **(Percent)**	
**Spine**	Experimental	39 (68.4)	18 (31.6)	0.02
	Control	52 (86.7)	8 (13.3)	
**Hip**	Experimental	53 (93)	4 (7)	0.65
	Control	57 (95)	3 (5)	

**Table 3:** Comparison of Bone Density in Spine and Hip Bones in Patients in Both Experimental and Control Groups

**Group** **Bone**	**Experimental **	**Control**	**P-value of Independent T-Test**
	**Mean±SD**	**Mean ± SD**	
**Spine**	-1.1±50.07	-0.1±78.20	0.001
**Hip**	-0.0±63.94	-0.0±33.98	0.09

**Table 4:** Comparison of Relative Frequency of Bone Density Reduction in Spine and Hip Bone in Hepatitis B Patients According to Risk Factors

**Bone** **Variable**	**Spine**		**Hip**	
	**Normal**	**Decrease of Bone Density**	**Normal**	**Decrease of Bone Density**
	**Number (Percent)**	**Number (Percent)**	**Number (Percent)**	**Number (Percent)**
**Age**	25 Years Or Less	12 (80)	3 (20)	14 (93.3)	1 (6.7)
	25-36 Years Old	12 (70.6)	5 (29.4)	17 (100)	0 (0)
	Over 35 Years Old	15 (60)	10 (40)	22 (88)	3 (12)
	P-value Chi-Square Or Fisher Test	^*^ 0.41	^**^0.36
**Body Mass Index**	Skinny	5 (50) 5 (50)	5 (50) 5 (50)	7 (70) 7 (70)	3 (30) 3 (30)
	Normal	24 (643-.9)	13 (35.1)	36 (97.3)	1 (2.7)
	Over-Weighted	10 (100)	0 (0)	10 (100)	0 (0)
	P-value Chi-Square Or Fisher Test	^**^0.03	**^**^0.40
**Hemophilia Severity**	Mild And Moderate	31 (70.5)	13 (29.5)	42 (95.5)	2 (4.5)
	Severe	8 (61.5)	5 (38.5)	11 (84.6)	2 (15.4)
	P-value Chi-Square Or Fisher Test	**^**^0.74	^**^0.22
**VitaminD**	20 And Less	16 (72.7)	6 (27.3)	21 (95.5)	1 (4.5)
	Over 20	23 (65.7)	12 (34.3)	32 (91.4)	3 (8.6)
	P-value Chi-Square Or Fisher Test	^*^0.58	^**^1.00
**Hepatitis**	No	32 (71.1)	13 (28.9)	42 (93.3)	3 (6.7)
	YES	7 (68.3)	5(41.7)	11 (91.7)	1 (8.3)
	P-value Chi-Square Or Fisher Test	**^**^0.49	**^**^1.00
**Smoking**	No	34 (66.7)	17 (33.3)	47 (92.2)	4 (7.8)
	YES	5 (83.3)	1 (16.7)	6 (100)	0 (0)
	P-valueChi-Square Or Fisher Test	**^**^0.65	**^**^1.00

*: Chi-Square **:Fisher Exact Test

**Table 5:** Regression Analysis of Bone Density Reduction in Spine and Hip Bone

**Predictive variables**		**B**	**SE**	**Beta **	**T**	**P**
**Spine bone**	Constant value	72.45	8.21	--	8.81	>0.005
	Age	-0.36	0.15	-0.3	-2.44	0.01
	BMI	1.09	0.35	0.4	3.05	0.004
	Vitamin D	-0.004	0.07	-0.006	-0.04	0.96
		ADJR^2^:0.13 R:0.42 R^2^: 0.18
**Hip bone**	Constant value	2.09	0.35	--	5.86	>0.005
	Age	0.01	0.007	0.36	2.89	0.006
	BMI	-0.06	0.01	-0.4	-3.9	>0.005
	Vitamin D	-0.001	0.003	-0.04	-0.36	0.71
		ADJR^2^:0.21 R:0.51 R^2^: 0.26
